# The bifurcation and topography of the posterior tibial artery within the tarsal tunnel

**DOI:** 10.1007/s00276-023-03115-w

**Published:** 2023-03-13

**Authors:** B. Marchese, A. McDonald, H. McGowan

**Affiliations:** Department of Microbiology, Anatomy, Physiology and Pharmacology, La Trobe University, Bundoora, VIC 3086 Australia

**Keywords:** Posterior tibial artery, Iatrogenic injury, Tarsal tunnel syndrome

## Abstract

**Purpose:**

The tarsal tunnel (TT) is a fibro-osseous anatomical space coursing from the medial ankle to the medial midfoot. This tunnel acts as a passage for both tendinous and neurovascular structures, including the neurovascular bundle containing the posterior tibial artery (PTA), posterior tibial veins (PTVs) and tibial nerve (TN). Tarsal tunnel syndrome (TTS) is the entrapment neuropathy that describes the compression and irritation of the TN within this space. Iatrogenic injury to the PTA plays a significant role in both the onset and exacerbation of TTS symptoms. The current study aims to produce a method to allow clinicians and surgeons to easily and accurately predict the bifurcation of the PTA, to avoid iatrogenic injury during treatment of TTS.

**Methods:**

Fifteen embalmed cadaveric lower limbs were dissected at the medial ankle region to expose the TT. Various measurements regarding the location of the PTA within the TT were recorded and multiple linear regression analysis performed using RStudio.

**Results:**

Analysis provided a clear correlation (*p* < 0.05) between the length of the foot (MH), length of hind-foot (MC) and location of bifurcation of the PTA (MB). Using these measurements, this study developed an equation (MB = 0.3*MH + 0.37*MC – 28.24 mm) to predict the location of bifurcation of the PTA within a 23° arc inferior to the medial malleolus.

**Conclusions:**

This study successfully developed a method whereby clinicians and surgeons can easily and accurately predict the bifurcation of the PTA, to avoid iatrogenic injury that would previously lead to an exacerbation of TTS symptoms.

## Introduction

### Anatomy of the tarsal tunnel

**Fig. 1:**
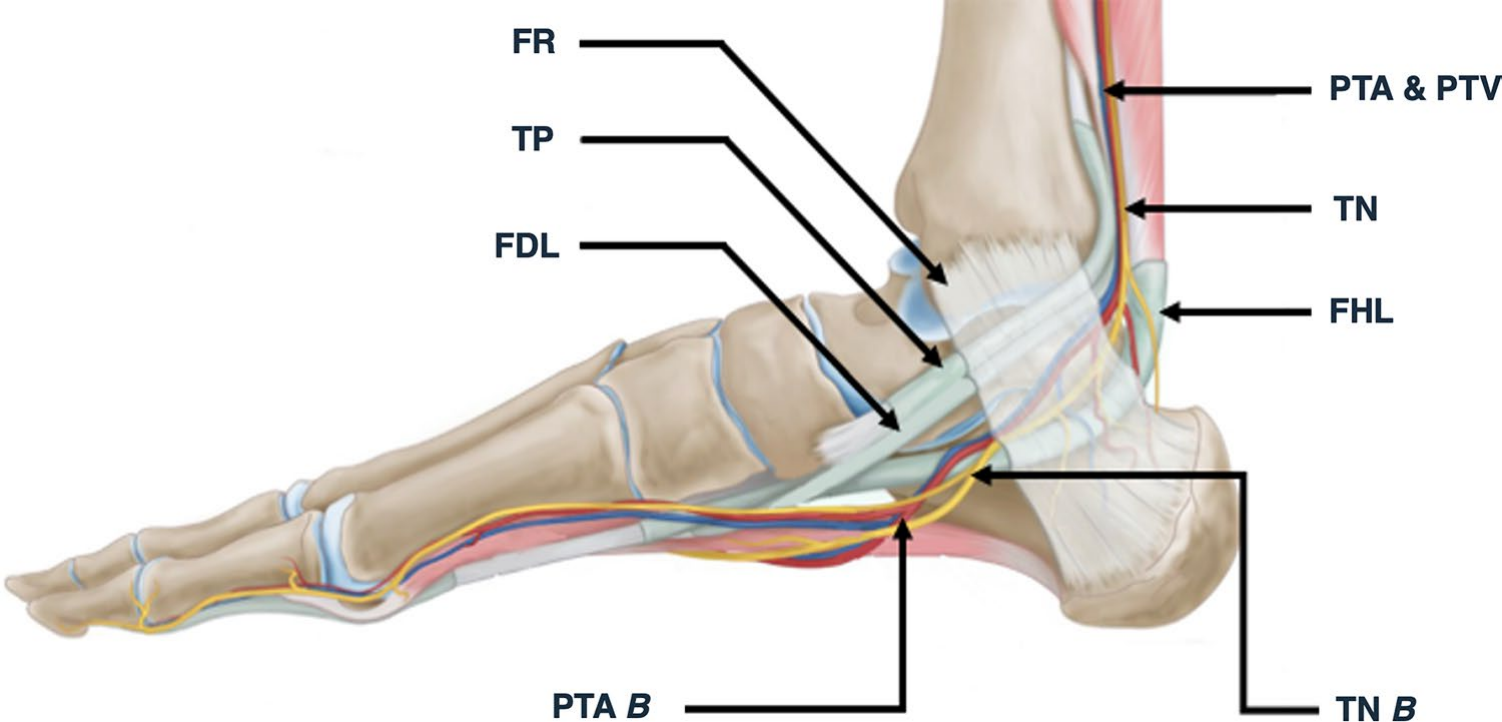
Medial view of the ankle joint presenting the contents of the tarsal tunnel; tibialis posterior tendon (TP), flexor digitorum longus tendon (FDL), posterior tibial artery (PTA), posterior tibial vein (PTV)*,* tibial nerve (TN) and the tendon of flexor hallucis longus (FHL), deep to the flexor retinaculum (FR). The bifurcation (*B*) points of the PTA (PTA *B*) and TN (TN *B*) are also represented. Adapted from [30]

The tarsal tunnel (TT) is a fibro-osseous anatomical space coursing from the medial ankle to the medial midfoot [2, 16, 18, 20]. This anatomical tunnel acts as a passage for both tendinous and neurovascular structures proceeding from the deep posterior compartment of the leg into the plantar aspect of the foot and heel [2, 16, 18, 20] (Fig. 1).

**Fig. 2:**
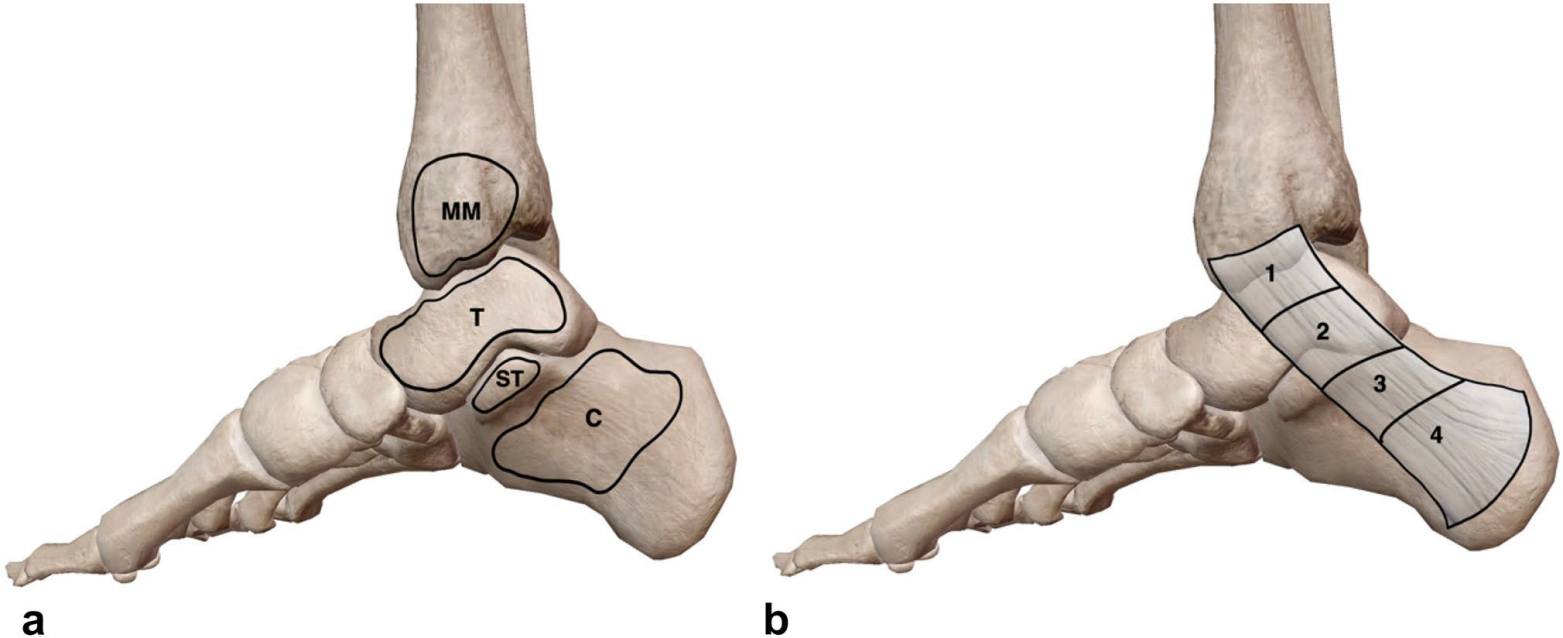
Medial view of the right ankle presenting the boundaries of the tarsal tunnel. **A** Representation of the bony aspects of the medial ankle that contribute to the borders of the tarsal tunnel; medial surface of the body of the calcaneus **C**, sustentaculum tali (ST), medial surface of the body of the talus (T) and the medial malleolus (MM). **B** black outlining the flexor retinaculum (roof of tarsal tunnel), with the addition of the three fibrous septa extending from this retinaculum running to the underlying bone to provide 4 fibrous canals (1, 2, 3, 4). Adapted from [34] (colour figure online)

The TT is bound anterosuperiorly by the medial malleolus, while the osseous floor is comprised of the medial surface of the body of talus and medial surface of the body of calcaneus including the sustentaculum tali [2, 16, 18, 20, 35] (Fig. 2a). The flexor retinaculum forms the roof of this anatomical space as well as encompassing its upper and lower boundaries [4, 18, 20, 35] (Fig. 2b). This retinaculum is comprised of two layers; superficial and deep. The deep layer extends three fibrous septa that attach onto the periosteum of the medial surface of the body calcaneus, separating the TT into four defined fibrous canals [18, 20] (Fig. 2b).

The TT allows flexor tendons and the neurovascular bundle that originate in the deep posterior compartment of the leg, to travel into the plantar surface of the foot and posterior heel [2, 18, 20]. Thus, the contents of the TT consist of the tendon of tibialis posterior contained within the first most medial fibrous canal, followed by the tendon of flexor digitorum longus located in the second canal. The neurovascular bundle containing the PTA, PTVs & TN is located lateral to these two tendinous structures and are all collectively contained within the third fibrous canal. Situated most laterally within the TT is the tendon of flexor hallucis longus which is contained within the fourth canal [2, 18, 20, 35] (Figs. 1, 2b).

### Anatomy of the posterior tibial artery

The PTA originates from the termination of the popliteal artery at the inferior border of the popliteus muscle, and then passes through the tendinous arch of soleus to enter the posterior compartment of the leg [21]. This vascular structure descends between the superficial and deep muscle compartments of the leg alongside the TN, supplying the posterior muscle compartments [6, 21]. The PTA then courses inferoposterior to the medial malleolus and enters the TT through the third fibrous canal alongside the TN and PTVs (Fig. 1). It is here that both the PTA & PTVs are located superficially and anteriorly to the TN [9, 21, 35]. Within the TT, proximal to its termination, the PTA gives rise to the medial calcaneal artery which acts as the blood supply for the heel and its associated fat pad [9]. The PTA will then terminate within the TT into the medial and lateral plantar arteries, generally distal to that of the bifurcation of the TN [6, 7, 9, 17, 21, 35]. The medial and lateral plantar arteries travel into and supply structures within the plantar surface of the foot [21] (Fig. 1).

### Tarsal tunnel syndrome

The entrapment neuropathy that describes compression, irritation and subsequent damage to the TN or its associated branches within the TT is referred to as tarsal tunnel syndrome (TTS) [2, 16, 18, 20, 27]. The compact and confined nature of the TT makes its contents particularly vulnerable to compression by even the slightest anatomical or physiological complications that reduce the overall space within this tunnel [20].

Both intrinsic and extrinsic causative factors of TTS syndrome have been recognized. These factors can be broadly classified as trauma, space-occupying lesions or systemic disease, with trauma being the most common aetiology [2, 4, 16, 18, 20, 26]. Damage and irritation of the TN associated with TTS can result in the formation of altered nerve sensation, pain and muscle weakness in the plantar and posterior surfaces of the foot and heel [1–3, 16, 18, 20].

### Treatment of tarsal tunnel syndrome

Initially addressed by conservative treatments including physiotherapy, commonly in conjunction with analgesics and anti-inflammatory medications, persistent cases of TTS often require further intervention [2, 16, 18, 20]. Corticosteroid and local anaesthetic injections are frequently administered in hopes of eliminating symptoms and eradicating the need for further invasive surgical treatments. However, multiple injection procedures are discouraged due to the increased risk of iatrogenic injury to the structures within the tarsal tunnel, including the PTA, with only a small to moderate chance of eradicating symptoms [4, 16, 18, 20]. Failure to alleviate symptoms can result in the consideration of more invasive intervention techniques [20].

Tarsal tunnel decompression surgery refers to the surgical procedure involved in alleviating pressure off the TN through the removal of compressive tissue including scarring, space-occupying lesions or anatomical anomalies [2, 4, 14, 18, 20]. While surgery is only performed in resistant cases of TTS, the literature regarding the efficacy of surgical intervention is highly conflicting and unclear [2, 16, 20, 26]. Studies focusing on the outcome of tarsal tunnel release surgery report variable results. Some studies report success in up to 85 – 96% of patients [2, 16, 18, 20], while other literature states no apparent improvement in up to 44% of patients [4]. Failure of this surgery can be associated with an inadequate release of the TN and its terminal branches throughout the proximal and distal aspects of the TT, as well as the failure to address perineural scarring [13, 20]. A major cause for failure of this surgery is iatrogenic injury of the TN or PTA due to overall inadequate anatomical knowledge of the region [13, 26].

### Risk of iatrogenic injury to the posterior tibial artery

Iatrogenic injury to the PTA can impact the outcome of both conservative and surgical procedures. Vascular trauma and haemorrhage of the PTA during these procedures can lead to scar formation within the TT and thereby, result in further compression of the TN [4, 13, 16, 18, 26]. Considering this, a comprehensive knowledge of the PTA’s anatomical pattern and pathway is integral to not only the understanding of the aetiology of TTS but is also paramount in the delivery of successful conservative and surgical treatments [9, 13, 32]. It is vital that clinicians and surgeons alike are knowledgeable regarding the PTA’s topography and bifurcation, to ultimately avoid iatrogenic injury during treatment and, furthermore, avoid further exacerbation of TTS symptoms [9, 13, 32].

### Literature regarding the location posterior tibial artery

The role that the PTA plays in both the formation and exacerbation of TTS is persistent and prominent in current literature [4, 16, 18, 20, 26, 29]. Despite this, literature regarding the anatomical pattern and pathway of the PTA is sparse [9, 17, 23, 32, 33, 35], especially when compared to that of the TN [8, 9, 14, 17, 24, 27, 31]. In addition, the few studies that do investigate this vascular structure [9, 17, 23, 32, 33, 35] demonstrate various inconsistencies in their use of specific bony landmarks and reference lines, the exact measurement parameters, presentation of results and explanations of dissection protocols and techniques. In addition, no study has examined the effect of the overall length of the foot on the location of the PTA.

Considering the role that the PTA plays in TTS, consistent and reproducible data regarding the anatomy of the PTA is vital. These data would not only enhance the understanding of the aetiology of this compressive neuropathy but would reduce iatrogenic injury to this vascular structure during both conservative and surgical treatment.

### Aims and hypothesis

In the light of previous literature, this study aims to develop a simplistic and reproducible approach to locate the PTA within the TT, relative to various anatomical structures. In addition, this study aims to provide a method whereby clinicians and surgeons alike can easily and accurately predict the location of the PTA to avoid iatrogenic injury during treatment of TTS.

This study hypothesizes that there is a relationship between the length of the foot and the location of bifurcation of the PTA within the TT. In addition, we also hypothesize that the use of a novel angle-based technique in relation to measurements regarding the PTA will be a more simplistic and representative method to apply in a clinical or surgical setting.

## Materials and methods

### Cadaveric specimens

Fifteen embalmed cadaveric lower limbs, stored in 10% ethanol, were dissected at the medial ankle region to expose the deep structures of the tarsal tunnel (TT). Seven right and eight left lower limbs were dissected, with 33% of specimens being female. The average age of death of cadaveric specimens investigated was 79 ± 1.7 years of age (68–91) and no vascular conditions were recorded in the donor documents. All cadaveric material was donated to La Trobe University through the Melbourne University Body Donor Program. Use of cadaveric specimens at La Trobe University is in conjunction with University of Melbourne Human Research Ethics Committee (Program No. 1544576.1/2015) with all methods and protocols aligning with the Human Tissues Act 1983.

### Foot placement and markings

**Fig. 3:**
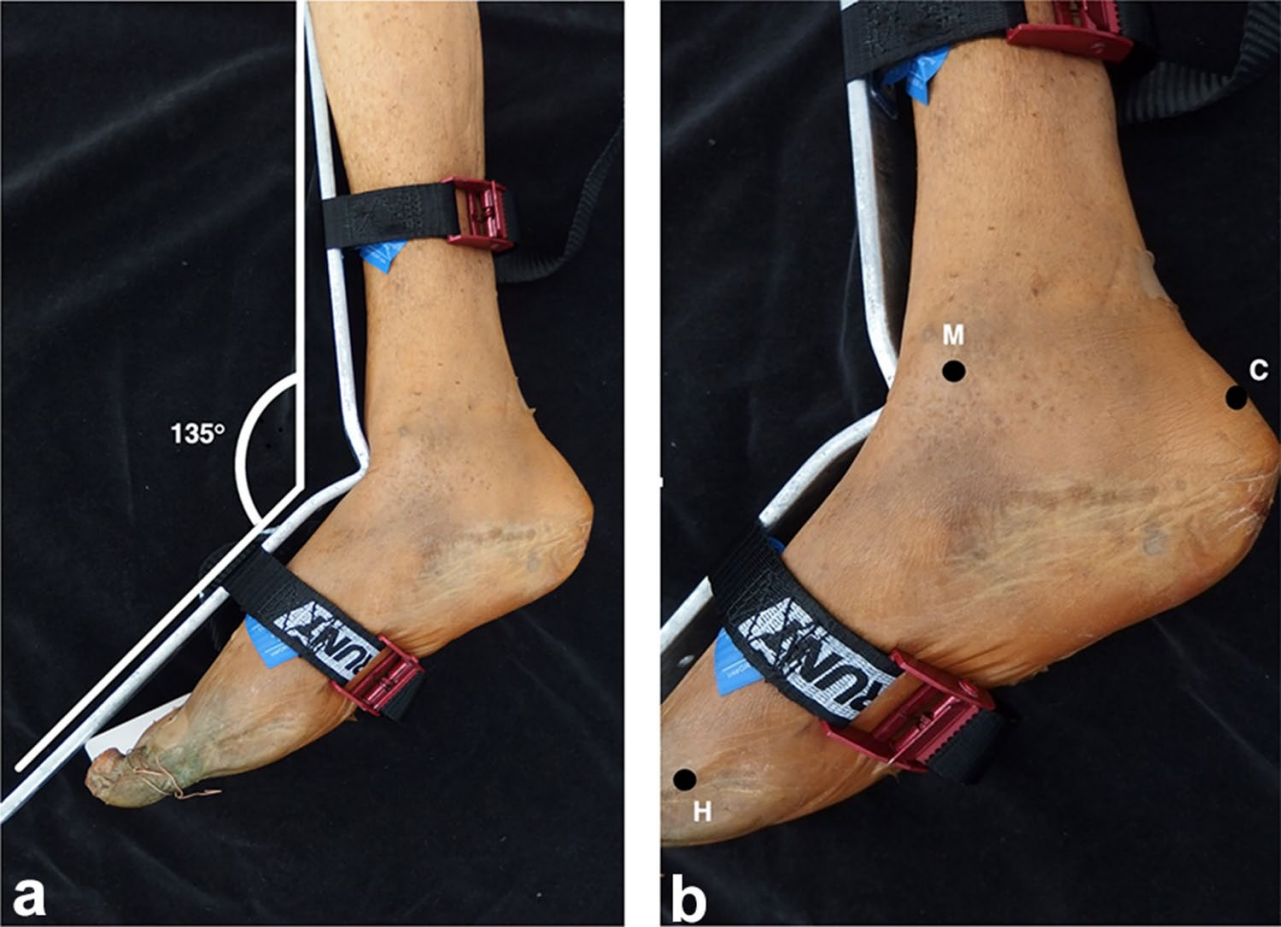
Medial view of right leg and foot. **A** 135° brace clamped to anterior leg, ankle & foot, to ensure maintenance of consistency when dissecting the tarsal tunnel. **B** zoomed image of photograph A, to show superficial anatomical bony landmarks used for measurements: inferior aspect of the medial malleolus (M), posterior superior aspect of the calcaneus **C** and head of the first metatarsal (H)

To establish consistency, cadaveric specimens were secured to a 135° ankle brace and positioned to have the medial ankle facing upright (Fig. 3a). Surface anatomical landmarks were palpated and marked. These included the most inferior tip of the medial malleolus (M), the posterior superior aspect of the calcaneal tuberosity (C) and the medial aspect of the head of the first metatarsal (H) (Fig. 3b).

**Fig. 4:**
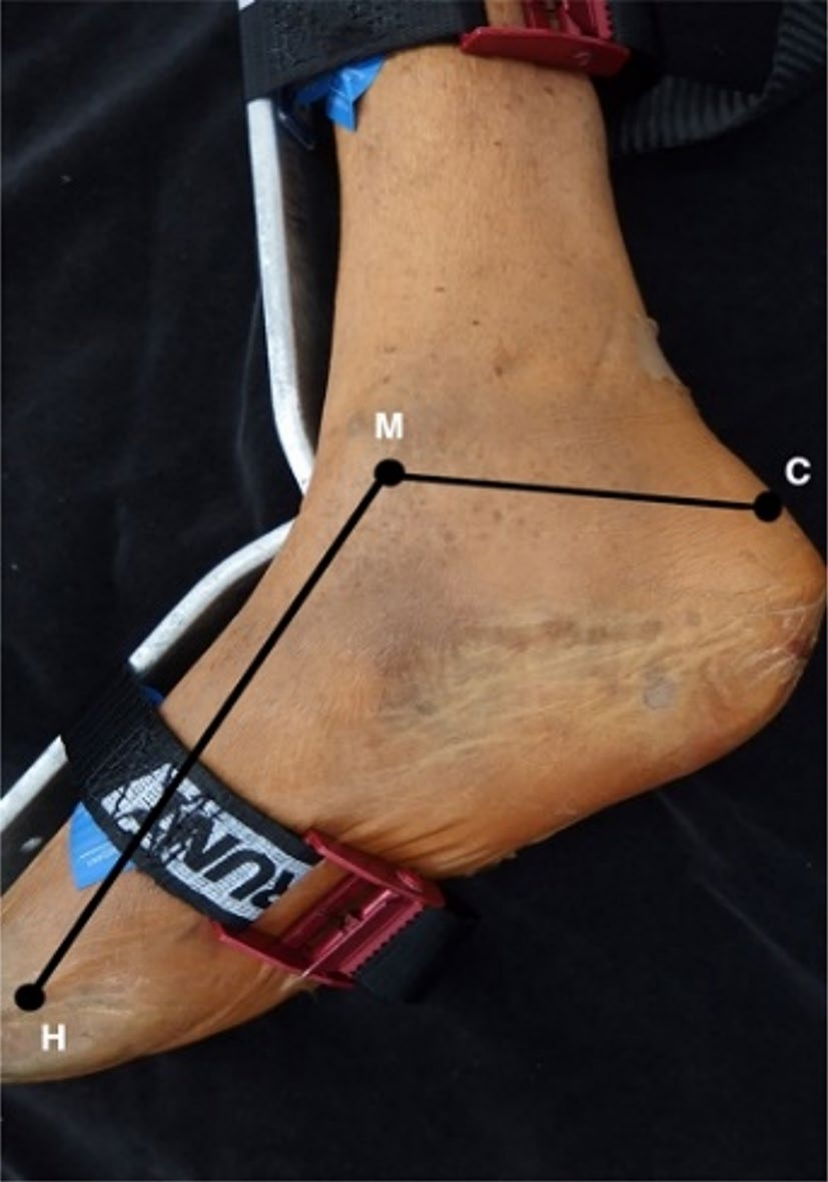
Medial view of the right leg and foot. Length of the hind-foot represented by the M–C (medial malleolus–calcaneus) axis and length of the foot represented by the M–H (head of the first metatarsal) axis

Following the marking of relevant anatomical landmarks, superficial measurements were recorded, including the distance between M and C, representing the length of the hind-foot, and the distance between M and H, being representative of foot length (Fig. 4). To allow for further measurement classification, an axis between M and C was devised and was termed the M–C axis which was set at 0°.

### Exposure of neurovascular structures

**Fig. 5:**
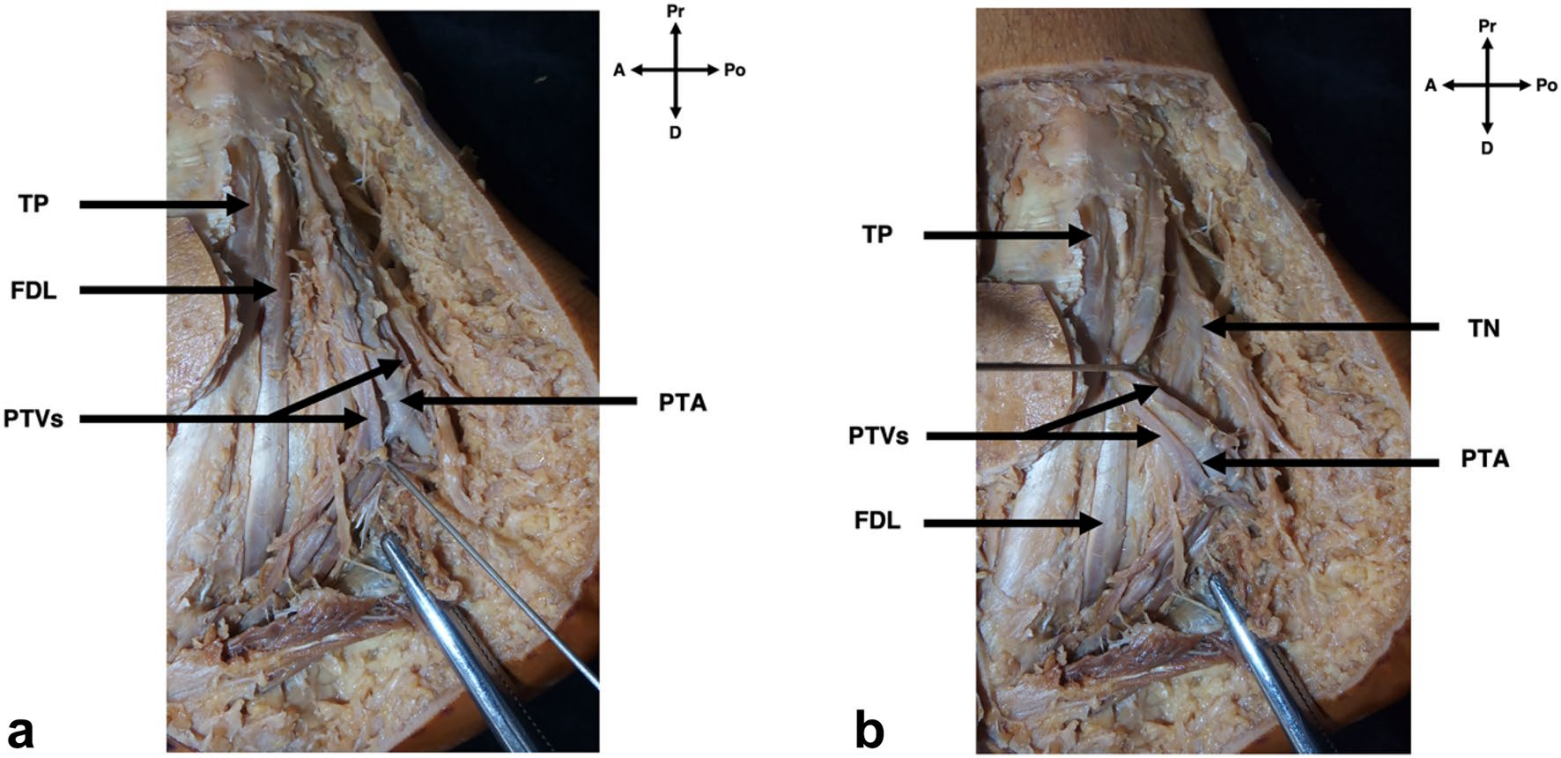
Exposed contents of the tarsal tunnel at the medial ankle. **A** Following removal of the flexor retinaculum and neurovascular sheath, the posterior tibial veins (PTVs) and posterior tibial artery (PTA) can be identified, as well as the tendon of tibialis posterior (TP) & the tendon of flexor digitorum longus (FDL). **B** Same specimen as 6A; however, the posterior tibial veins and posterior tibial artery have been retracted to locate the tibial nerve (TN), (compass: *A* Anterior, *Po* Posterior, *Pr* Proximal, *D* Distal)

Dissection began with the removal of skin and adipose tissue of the medial ankle region, resulting in the exposure of the flexor retinaculum. After the removal of the flexor retinaculum, the structures of the tarsal tunnel were clearly visible within their own fibrous canals, separated by thick fibrous septa as supported by previous literature [18, 20]. The contents beginning most medially were the tendon of tibialis posterior, the tendon of flexor digitorum longus, the neurovascular bundle encased in neurovascular sheath, containing the PTA, PTVs and the TN, and most laterally the tendon of flexor hallucis longus (Fig. 5a, b). To accurately locate the bifurcation of the PTA, the neurovascular sheath encasing this neurovascular bundle was removed, although only where necessary to preserve the PTA’s natural position within the TT.

### Measurement of neurovascular structures

**Fig. 6:**
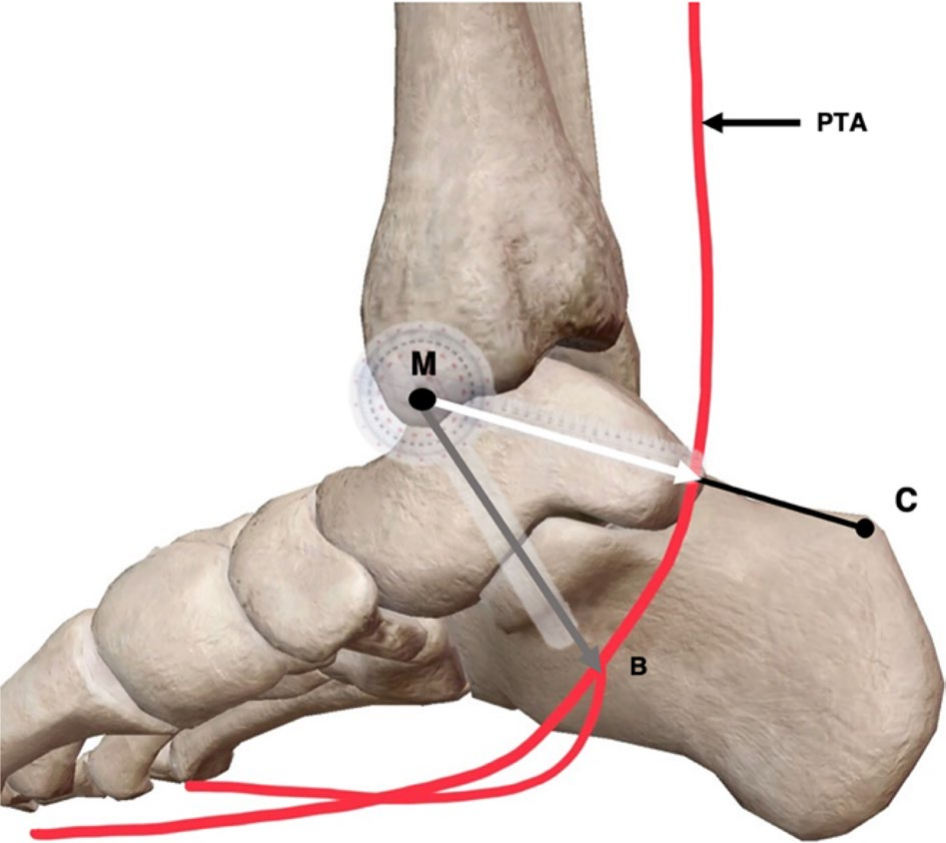
Medial view of the right ankle and foot presenting measurement methodology regarding the posterior tibial artery. Ankle braced at 135° angle. Location of the main trunk of the posterior tibial artery (PTA) along the M–C (medial malleolus–calcaneus) as a distance from M (white arrow). Location of bifurcation (B) of the posterior tibial artery as a distance from M (grey arrow). Angle of bifurcation of the PTA from the M–C (black arrow), adapted from [34] (colour figure online)

Following exposure of the neurovascular bundle, data regarding both the PTA’s bifurcation and topography were recorded. Factors regarding the general topography of the PTA included the artery’s depth relative to the TN, and the anatomical position of the PTA compared to the TN. In addition, the location of bifurcation of the PTA with respect to the TN was recorded. Following this, the location of the main trunk of the PTA along the M–C axis was recorded by measuring the distance from M to the location of the PTA along this axis (Fig. 6).

The location of PTA bifurcation was then recorded by measuring the distance from M to the exact location of bifurcation (B), this was referred to as the M–B length (Fig. 6). Lastly, the angle of bifurcation from the M–C axis was measured. If the angle of bifurcation was proximal to the M–C axis it was assigned a negative value, while an angle distal to this axis was represented through a positive value (Fig. 6).

After the completion of these measurements, the ankle was released from the 135° brace. For comparison of results and observation of the effect of ankle angle, the ankles of five randomly allocated specimens were then attached to a 90° ankle brace. The previously stated measurements were then completed at this angle and recorded.

### Data analysis

All data were entered into RStudio in which the 3Dscatterplot package was downloaded and installed [25]. Multiple linear regression analysis was undertaken to correlate the distance of PTA bifurcation (B) from the inferior tip of the medial malleolus (M), with respect to the length of the foot (M–H) and the expected angle of bifurcation. Student’s *t* tests were performed in GraphPad Prism to analyse any relevant changes in data between 135° and 90° ankle placement.

## Results

### Introduction

The following sections will detail results regarding the bifurcation and topography of the PTA within the TT. For this current study, results discussed will be relative to the ankle braced at a 135° angle, with length measurements presented in the form of millimeters (mm) ± standard error of the mean (SEM), unless specified otherwise.

### General anatomy and topography of the posterior tibial artery

The PTA in all cases, bifurcated into the medial and lateral plantar arteries distal to the M–C axis. As expected, the PTA was located within the third fibrous canal of the TT, along with the TN and PTVs (Fig. 5a, b).

### Relationship of the posterior tibial artery relative to the tibial nerve

As iatrogenic injury of the PTA can directly impact the outcome of both conservative and surgical treatment [16, 18, 26], the current study felt that it was important to report on the topography of the PTA, specifically in relation to the TN. As represented in Fig. 5A, B, the main trunk and terminal branches of the PTA in all specimens were situated superficial and anterior to the TN. In addition, the PTA bifurcated in every case distal to the bifurcation of the TN.

### Foot size and reference line dimensions

**Fig. 7:**
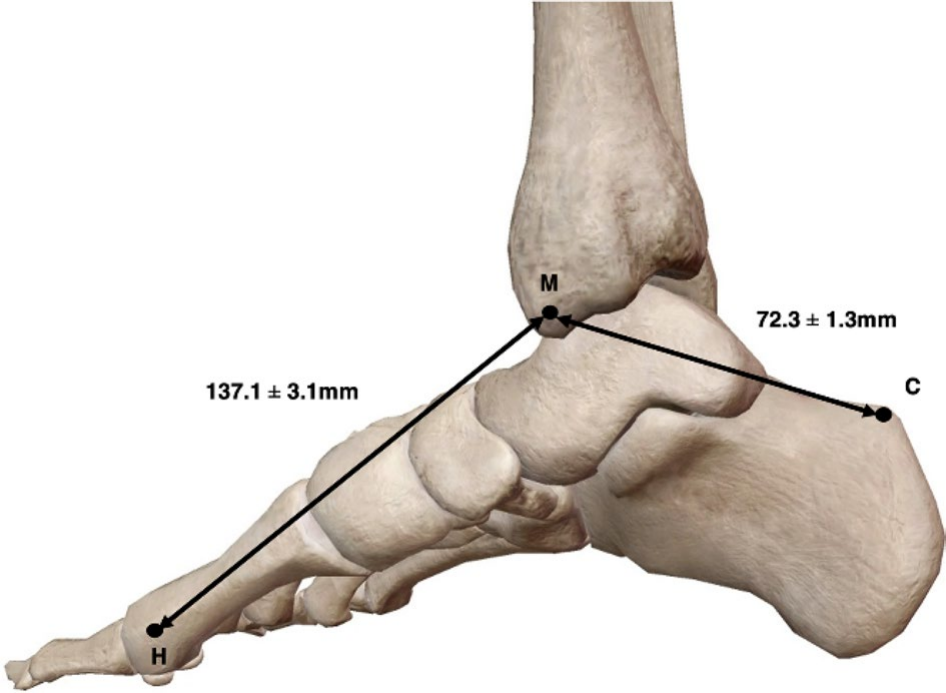
Medial view of the right ankle and foot presenting average foot and hindfoot length. Average length of the foot measured as a distance between M (medial malleolus) and H (head of the first metatarsal): 137.1 mm ± 3.1 mm, while the average hind-foot length measured as a distance between M and C (calcaneus): 72.3 mm ± 1.3 mm. All measurements represented in the form of mm ± standard error of the mean, Adapted from [34]

Data collection to identify variation in foot size was deemed an important factor to include when determining location of the PTA. The average length of the hind-foot or M–C length in this study was 72.3 ± 1.3 mm, while the mean M–H length, which was used to represent the length of the foot, measured 137.1 ± 3.1 mm (Fig. 7).

### Location of the posterior tibial artery along the M–C axis

**Fig. 8:**
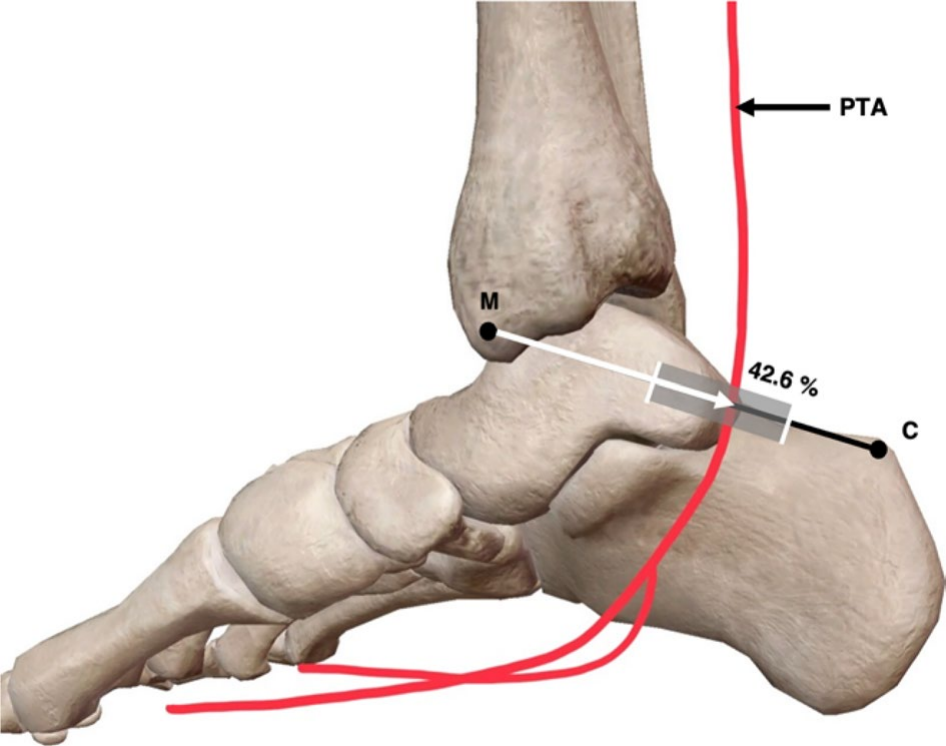
Medial view of the right ankle and foot presenting location of posterior tibial artery. The posterior tibial artery (PTA) crossed the M–C (medial malleolus–calcaneus) axis on average at 42.6% ± 1.6% of its length. In addition the range, represented in grey rectangle, of the M–C axis, at which the PTA of all specimens in this study crossed: 35.5–58.2% of its length, measured as a distance from M. Adapted from [34] (colour figure online)

Although this study primarily focused on the bifurcation of the PTA, details about the location of the main trunk of this vascular structure were also recorded. The main trunk of the PTA crossed the M–C axis at a range between 35.5% (25.7 mm) and 58.2% (42.1 mm) of the M–C axes length, with it crossing this axis on average at 46.2 ± 1.6% (33.5 ± 1.5 mm) from M (Fig. 8).

### Angle of bifurcation of the posterior tibial artery

**Fig. 9:**
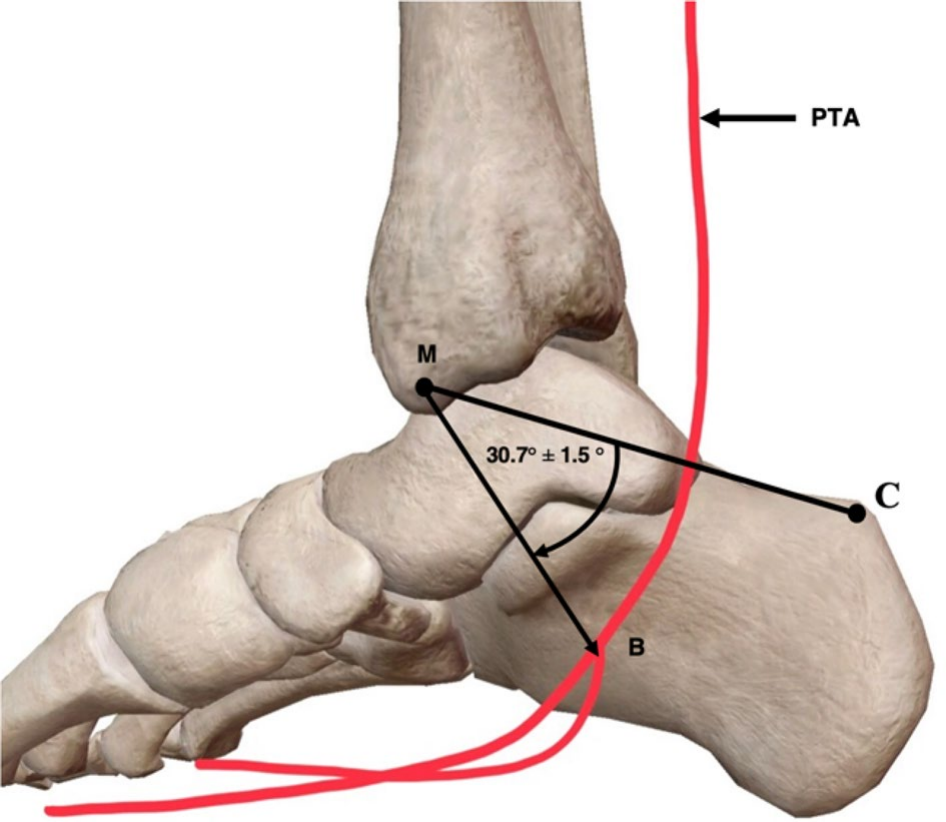
Medial view of the right ankle and foot representing angle of posterior tibial artery bifurcation. Average angle (30.7°) that the posterior tibial artery (PTA), bifurcated (B), from the M–C axis (medial malleolus–calcaneus). Adapted from [34]

The angle of bifurcation of the PTA was a novel measurement technique employed in this study, primarily chosen for its clinical applicability. In this study, the average angle at which the PTA bifurcated from (distal) the M–C axis was 30.7° ± 1.5° (Fig. 9).

### Danger zones

**Fig. 10:**
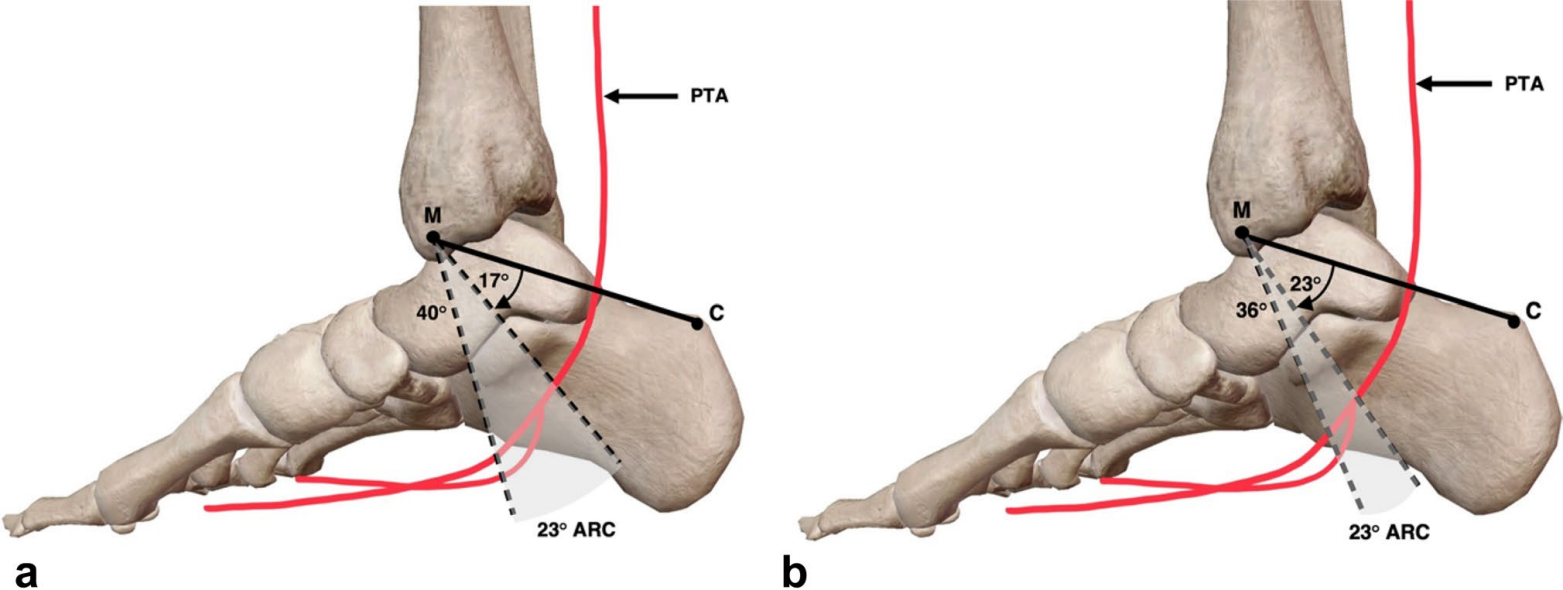
Medial view of the right ankle and foot presenting ‘danger zones’. **A** Bifurcation of the posterior tibial artery (PTA) in all specimens, occurred within a 23° zone between 17 and 40° distal to that of the M–C (medial malleolus–calcaneus). **B** 87% (13/15) of specimens in this study showed a PTA that bifurcated within a 13° zone from 23° to 36° distal to M–C. Adapted from [34]

Further investigation into the angle of bifurcation of the PTA revealed a pattern of bifurcation locations. This analysis resulted in the development of 23° and 13° ‘danger zones’, which characterize the PTA’s most likely bifurcation position within the TT (Fig. 10a, b). These ‘danger zones’ were presented in the forms of arcs to ensure the curved nature of the tarsal tunnel was adequately accounted for.

In all cases in this study, the PTA bifurcated within a 23° zone between 17° and 40° distal to that of the M–C axis (Fig. 10a). Furthermore, 87% (13) of cadaveric specimens dissected exhibited a PTA that bifurcated within a 13° zone between 23° and 36° distal to that of M–C axis (Fig. 10b). A multiple linear regression analysis revealed that the angle of bifurcation is not significantly correlated to the length of the foot or hind-foot, ultimately suggesting that the angle of bifurcation of the PTA is not influenced by the length of the foot. This more importantly indicates that the danger zones devised in this study will remain constant across various foot lengths, as they are independent of foot size.

### Influence of ankle position

Five cadaveric specimens were randomly chosen to determine whether ankle angle influenced the location of PTA bifurcation. Selected specimens were subjected to measurements as described in the above methods; however, ankles were braced at a 90° rather than 135° A paired *t* test was used to compare the average angle of bifurcation in 135° and 90° ankle settings. As presented in Table 1, there is a statically significant difference in the average angle of bifurcation when the ankle is braced at 135° compared to that of 90° (*p < 0.05). These results indicate that as the ankle dorsiflexes, the PTA will slide towards the proximal end of the TT. More simply put, the bifurcation of the PTA will be located more proximal in a dorsiflexed position, compared to that of a relaxed ankle position.

**Table 1:** Ankle angle influence. The average angle of posterior tibial artery bifurcation from M–C in 135° and 90° ankle angle settings, (*n* = 5) *p < 0.05

	Measurements (°)	
	Ankle braced at 135°	Ankle braced at 90°
Average angle of bifurcation from M–C	32 ± 3.7	28 ± 4.4*

### Location of bifurcation of the posterior tibial artery

RStudio 3Dscatterplot function was used to complete a multiple linear regression analysis. This analysis provided a statistically significant (*p < 0.05) linear correlation between the length of the foot (M–H*), and the distance of PTA bifurcation from the most inferior aspect of the malleolus (designated M–B), as well as establishing a correlation with the length of the hind-foot (M–C).

The equation produced states that the distance of bifurcation of the PTA from M will be as follows; *M–B(mm)* = *0.3(M–H)* + *0.37(M–C) – 28.24.* This analysis indicated a significant correlation between length of the foot (M–H), and the location of bifurcation (M–B). Simply put, the longer the foot length (M–H), the more inferior the PTA bifurcation relative to the medial malleolus (M–B). The coefficient of determination (R^2^) of the equation produced was R2 = 0.4. In random biological systems, an R^2^ equal to 0.4 indicates a substantial correlation [19]. In this study, this value indicates that the length of the foot (M–H) and the location of PTA bifurcation (M–B) are substantially correlated. RStudio input commands and data outputs can be located in the appendix.

## Discussion

### Introduction

Tarsal tunnel syndrome (TTS) is an entrapment neuropathy that describes the symptomatic distress related with compression, irritation and damage of the tibial nerve (TN) within the tarsal tunnel (TT) [1, 2, 16, 18, 20]. Iatrogenic injury to the posterior tibial artery (PTA) during both conservative and surgical treatment of TTS can potentially lead to compression of the TN and further exacerbation of this entrapment neuropathy [4, 16, 18, 26]. Despite the prominent involvement of this vascular structure in the exacerbation of TTS, literature regarding the general topography of the PTA within the TT is limited [9, 17, 23, 32, 33, 35], especially when compared to that of the TN [8, 9, 14, 17, 24, 27, 31]. In addition, no published study (to the authors’ knowledge) has considered the length of the foot, and its effect on the location of PTA within the TT. Considering this, the current study was able to demonstrate a relationship between the length of the foot and the location of bifurcation of the PTA. Furthermore, this study was able to provide simple instructions for a clinician or surgeon to confidently predict the bifurcation of the PTA within the TT using a novel angle-based technique, to help avoid iatrogenic injury and improve TTS treatment outcomes.

### Location of bifurcation of the posterior tibial artery

**Fig. 11:**
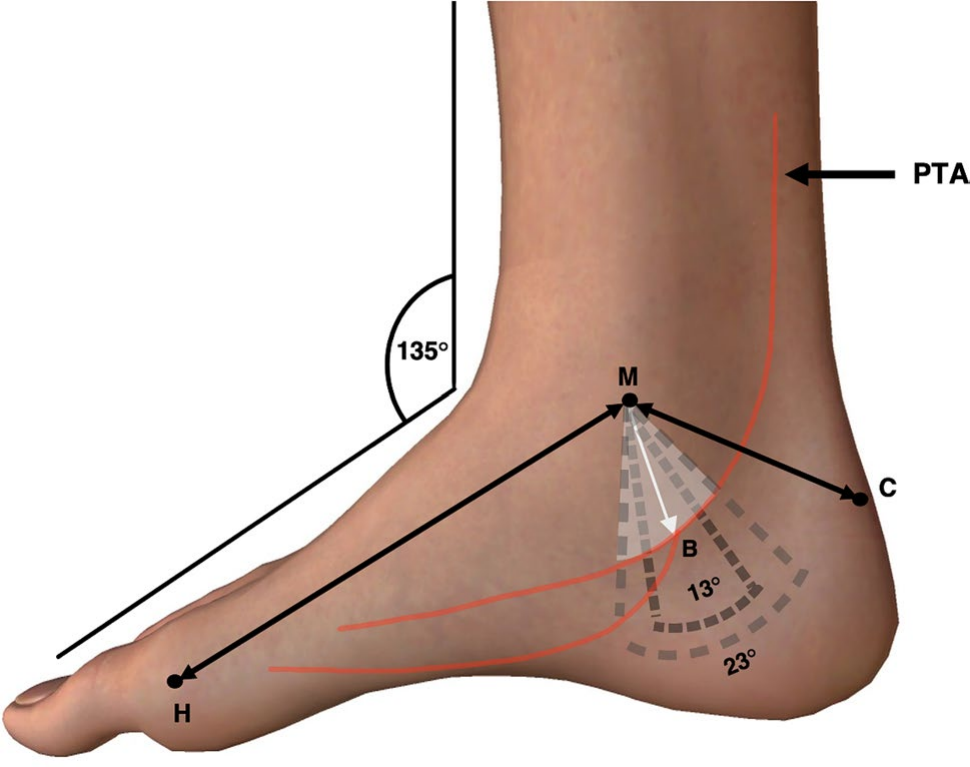
Medial view of the right ankle and foot presenting clinical applications of results. Clinicians can measure between 17° and 40° distal to the M–C (medial malleolus – calcaneus) axis, to mark out a 23° danger zone. This zone indicates where posterior tibial artery (PTA) bifurcation will most likely occur. A 13° danger zone within this arc can be measured between 23° and 36° distal to the M–C axis, representing where 87% of specimens in this study presented a bifurcating PTA. Finally, as per calculated using the provided equation, the distance of PTA bifurcation from M can be marked within both the 23° and 13° danger zones. Adapted from [34]

As previously discussed, one major reason for iatrogenic injury to the PTA during TTS treatment is the difficulty in accurately determining its pathway through the TT [13, 26]. Literature suggests that there are variable methods employed to predict the location of this vascular structure, such as palpation of the artery, magnetic resonance imaging (MRI) or ultrasound, [5, 10, 12]. Firstly, although palpation can be performed to identify the PTA, it cannot be used to follow this along to determine where it bifurcates. Furthermore, MRI and ultrasound may not be readily available, especially for clinicians performing injection procedures. One of the main aims of this study was to provide a method whereby clinicians and surgeons can easily and accurately predict the location of the PTA, to avoid iatrogenic injury. We hope that these findings can be used in conjunction with other techniques to ensure minimal damage occurs and the treatment of TTS is as effective as possible, especially in cases where predictive techniques such as ultrasound are unavailable. To allow for the most clinically applicable results, this study has devised recommendations for clinicians and surgeons on how to apply our data to locate the PTA within the TT. The following discussion will provide step-by-step instructions on how to simply and reproducibly do this (Fig. 11).

First, clinicians or surgeons should pre-operatively brace or position the ankle at a 135° angle. This relaxed ankle position is consistent with that of surgical procedures, although ensuring this position is consistent with that of 135° angle will warrant that results from this study are applied as accurately as possible. Throughout the literature, the angle of the ankle was presented as either 90° [17, 23, 35], or was not reported at all [9, 24, 32]. As seen in our results, the location of PTA bifurcation varies significantly between ankle angles, suggesting that the location of this vascular structure is dependent on the angle in which the ankle is situated. Given the relaxed ankle position employed during clinical settings [11, 28], we strongly recommend further research and any treatment adopt the more natural angle of 135°.

Second, there are a variety of bony landmarks presented amongst the current literature [9, 17, 23, 32, 33, 35], with some ambiguity related to how identifiable these landmarks are in clinical settings. Various aspects of the medial malleolus of the tibia were described within previous literature. Some studies utilised the most inferior aspect [17, 23, 32], one study used the most prominent tip [35], while other studies did not specify [9, 33]. The medial malleolus is a prominent anatomical bony landmark and can be easily palpated [22] although it is a large structure with multiple places to use during measurements and clinical procedures. Therefore, this study suggests using the most inferior tip of the medial malleolus as it can be readily palpated with no ambiguity in locating it; therefore, it is ideal to be used in a clinical setting.

The calcaneal tuberosity is another large bony landmark which was regularly employed across other studies [9, 17, 23, 32, 33, 35]. While most studies utilized the posterior superior aspect of the calcaneal tuberosity [9, 17, 23, 33, 35], one study did not state the aspect that was utilized [32]. Similar to that of the medial malleolus, inconsistences or ambiguity regarding this relatively large bony landmark can lead to difficulties applying it clinically. Furthermore, dorsiflexion of ankle leads to obstruction of the posterior superior aspect of the calcaneal tuberosity due to the taught nature of the calcaneal tendon overlying it [21]. This study recommends the use of the posterior superior aspect of the calcaneus as it can be easily palpated although at a relaxed ankle angle to ensure its prominent nature remains unobstructed by the calcaneal tendon.

As discussed above, multiple variations in surface landmarks leads to inconsistences in replicating findings in a clinical setting. Importantly, this has the potential to cause iatrogenic injury to deeper structures such as the PTA. As injury to the PTA is one of the leading causes of exacerbating TTS, we propose that the following, easily identifiable bony landmarks be used for all following procedures requiring location of structures in the TT: the inferior tip of the medial malleolus, the posterior superior aspect of the calcaneus with the ankle situated at a relaxed angle, and the medial aspect of the head of the first metatarsal (Fig. 11).

Third, clinicians and surgeons should then measure the distance between the inferior tip of the medial malleolus and the posterior superior aspect of the calcaneus (M–C), and the inferior tip of the medial malleolus and the medial aspect of the head of the first metatarsal (M–H). M–C will represent the patients’ hind-foot length and M–H their foot length, which are then inputted into the derived equation discussed in following sections (Fig. 11). The M–C axis should then be set at 0° for further applications.

Clinicians should then locate ‘danger zones’ to identify the most probable location of the patient’s PTA bifurcation. To do this, clinicians should mark out a 23° arc by measuring 17°–40° distal to that of the M–C axis, which will represent the patients most likely position of PTA bifurcation. To increase specificity of this danger zone, a 13° arc can be measured between 23° and 36° distal to the M–C axis, which will represent an 87% chance of the patients PTA bifurcation location (Fig. 11). This study approached measurement of PTA bifurcation with a novel angle-based technique which was chosen for its simplicity and reproducibility, specifically when applied in a clinical setting. Prior studies that investigated the location of the PTA presented data and results through a variety of result presentation methods [9, 17, 23, 32, 33, 35]. The array of result presentation and variable methodology across these studies can make it difficult to come to a conclusion regarding the location of the PTA within the TT.

Similar previous literature [9, 17, 23, 32, 33, 35], axes in this current study were used as a general classification tool regarding the location of the PTA. Despite this, there were a variety of references lines employed across previous literature with each study measured alternative aspects of the PTA. Results produced in this study in relation to the location of the main trunk of the PTA along the M–C axis specifically, are consistent with findings from one study [17]. This study demonstrated that this vascular structure would cross the M–C line at 48% of its length, similar to the current study in which the PTA was located at 46.2% of the M–C axes length. Furthermore, the range at which the PTA crossed the M–C was similar in both the current study and past literature [17]. Although the placement of the main trunk of the PTA is not directly related to the method used to locate bifurcation in this study, the variance in results can complicate clinical application cause general confusion regarding the anatomy of the PTA within the TT.

The production of safe and danger zones relative to the PTA were examples of result presentation methods employed across two previous studies [17, 32]. Despite this, zones established across these two studies are relative to two separate regions of the PTA, with one study only considering the main trunk of the PTA when developing a safe zone [32]. In addition, the range of zone presentation ranged from linear to quadrant zones, which may make extrapolation of these results into a clinical setting difficult. Therefore, we suggest the use of angle-based arc ‘danger zone’ approach as it is simplistic can be effortlessly reproduced in a clinical setting, while adequately embody the curved nature of the TT.

The length of the foot is an essential variable to consider when trying to effectively locate neurovascular structures within the TT, including the PTA. The overall length or size of the foot was one of the most variable measurements recorded in this study, ranging from 113 to 159 mm. Despite this, prior studies investigating the PTA fail to consider the length of the foot, past that of the hind-foot length [9, 17, 23, 32]. As seen in the results section, this study was able to show that there was a significant correlation between the length of the foot and the location of PTA bifurcation. These results support the assumption that foot size is significant to PTA location and stress consideration of this factor to produce accurate results. In addition, results proved that there was not a significant correlation between the length of the hind-foot and the location of PTA bifurcation. Therefore, studies that employ this as a representation of foot length alone will not have represented foot length adequately.

Finally, using the patients foot length (M–H) and hind-foot length (M–C), the following equation should be applied; MB(mm) = 0.3(MH) + 0.37(MC) – 28.24, to calculate the distance of bifurcation (B) of the PTA from M (M–B) (Fig. 11). Clinicians and surgeons can now simply measure the calculated distance inferior to that of the medial malleolus, to the point of bifurcation of the PTA. This will enable the identification of the PTA within the TT, to overall help avoid iatrogenic injury of the structure.

### Limitations and future directions

First, the researchers acknowledge the importance of anatomical variation and anomalies when presenting these findings. Given there is no consensus on the general anatomy of the PTA and its terminal branches, this study focussed on this detail. Further investigation to build on this knowledge by including anomalies is strongly supported. Additionally, the authors are aware that the flexor digitorum accessories longus muscle has been located in the TT (2–14%; mainly in males; mainly unilaterally); however, this muscle was not identified in any specimens in this study. This accessory muscle has been shown to compress the TN [15], and may potentially alter the location of the PTA. Replicating this study using higher numbers of cadaveric specimens may be able to help fine-tune the location of danger zones, increasing accuracy in clinical use. Increased numbers may also help identify possible anomalies for a clinician or surgeon to be aware of. Furthermore, it is very difficult to source cadaveric specimens with an age variation representative of the general population; however, trying to increase age variations that representing those most prone to TTS, may also be beneficial. We would also like to mention that although TTS is more common in females [18, 20] with the age range is extremely variable [16, 20]. A general limitation of using embalmed cadaveric specimens is that they are fixed; therefore, they may be a less than accurate representation of the living population. To confirm the accuracy of results, future applications may include applying data to live cohorts to try to confirm the predicted location of PTA bifurcation through medical imaging techniques.

## Conclusions

The main aim of this study was to develop a simplistic and reproducible approach to locate the PTA within the TT. Using consistent bony landmarks, taking into consideration foot length and applying the equation developed in this study, we are confident that this aim has been achieved. More importantly, this study has successfully developed a method whereby clinicians and surgeons can easily and accurately predict the bifurcation of the PTA, to avoid iatrogenic injury that would previously lead to an exacerbation of TTS symptoms.

## Data Availability

Not applicable.

## RStudio commands

Install.packages (“scatterplot3d”) # Install

Library (“scatterplot3d”) # load

Library (readr)

Correlation_analysis <—read_csv (“correlation_analysis.csv”)

Model <—lm (MB ~ ,.,data = correlation_analysis)

Lm (formula = MB ~ , .,ata = correlation_analysis)

Summary (model)

## Outputs from RStudio

Call: lm (formula = MB ~ ,.,data = correlation_analysis).

Residuals:

Min 1Q Median 3Q Max.

 − 5.477 − 3.233 – 1.844 1.341 11.776

Coefficients:

Estimate Std. Error *t* value Pr ( >|t|).

(Intercept)  − 28.2366 23.17 − 1.219 0.2446.

MH 0.2952 0.1243 2.374 0.0337 *

MC 0.3722 0.3014 1.235 0.2388

Signif. codes: 0 ‘***’ 0.001 ‘**’ 0.01 ‘*’ 0.05 ‘.’ 0.1 ‘’ 1.

Residual standard error: 5.526 on 13 degrees of freedom.

Multiple *R*-squared: 0.4305, Adjusted R-squared: 0.3429.

*F* statistic: 4.913 on 2 and 13 DF, *p* value: 0.02575.

Therefore,

MB = 0.30*MH + 0.372*MC − 28.24.

*R*^2^= 0.43

*P* < 0.05.
